# Surgical management of perianal fistula using an ovine forestomach matrix implant

**DOI:** 10.1007/s10151-023-02809-y

**Published:** 2023-05-03

**Authors:** A. Hsu, K. Schlidt, C. R. D’Adamo, B. A. Bosque, S. G. Dowling, J. H. Wolf

**Affiliations:** 1 Department of Surgery, Sinai Hospital, Baltimore, MD USA; 2 University of Maryland School of Medicine, Baltimore, MD USA; 3 Aroa Biosurgery Limited, Auckland, New Zealand; 4 Department of Surgery, George Washington University, Washington, D.C., USA

**Keywords:** Ovine forestomach matrix, Anal fistula, Perianal fistulas, Extracellular matrix, Fistula plug

## Abstract

**Purpose:**

Invasive surgical management of cryptoglandular perianal fistulas (PF) is challenging because of high recurrence rates and the potential for injury to the sphincter complex. In the present technical note, we introduce a minimally invasive treatment for PF using a perianal fistula implant (PAFI) comprising ovine forestomach matrix (OFM).

**Methods:**

This retrospective observational case series highlights 14 patients who had undergone a PAFI procedure at a single center between 2020 and 2023. During the procedure, previously deployed setons were removed and tracts were de-epithelialized with curettage. OFM was rehydrated, rolled, passed through the debrided tract, and secured in place at both openings with absorbable suture. Primary outcome was fistula healing at 8 weeks, and secondary outcomes included recurrence or postoperative adverse events.

**Results:**

Fourteen patients underwent PAFI using OFM with a mean follow-up period of 37.6 ± 20.1 weeks. In follow-up, 64% (*n* = 9/14) had complete healing at 8 weeks and all remained healed, except one at last follow-up visit. Two patients underwent a second PAFI procedure and were healed with no recurrence at the last follow-up visit. Of all patients that healed during the study period (*n* = 11), the median time to healing was 3.6 (IQR 2.9–6.0) weeks. No postprocedural infections nor adverse events were noted.

**Conclusions:**

The minimally invasive OFM-based PAFI technique for PF treatment was demonstrated to be a safe and feasible option for patients with trans-sphincteric PF of cryptoglandular origin.

**Supplementary Information:**

The online version contains supplementary material available at 10.1007/s10151-023-02809-y.

## Introduction

Surgical management of cryptoglandular perianal fistulas (PF) is challenging because of high recurrence rates and the potential for injury to the sphincter complex [1]. Invasive procedures, such as ligation of intersphincteric fistula tract (LIFT) or mucosal advancements flaps, can offer greater clinical efficacy in comparison to less invasive methods, such as fibrin glue injection. There is a trade-off, however, in that more invasive methods also carry an increased risk of postoperative complications, including infection, bleeding, or anal sphincter damage [2]. Efforts to develop less-invasive methods with higher efficacy have included techniques such as video-assisted anal fistula treatment (VAAFT), fistula laser closure (FiLaC), and stem cell therapy [3]. While promising, these more contemporary options have their own unique challenges, e.g., high capital equipment cost, limited access to training opportunities, or complex programmatic needs for autologous stem cell transplantation [4].

In addressing PF closure, a need still exists for a minimally invasive and clinically efficacious alternate to traditional surgical interventions, which has led to investigations using regenerative biomaterials [1]. Biological implant materials, typically derived from animal or human tissues, have seen adoption in PF treatment [1]. Typically, these types of devices are used as a “fistula plug” to occlude openings to the fistula and are considered less invasive than traditional surgical approaches. However, while biomaterial-based fistula plugs have low patient risk, they also have traditionally demonstrated relatively low clinical efficacy when compared to invasive flap procedures [1]. The ideal biomaterial to serve as a fistula implant must tolerate bacterial contamination and counter local tissue inflammation. This led the authors to postulate that ovine forestomach matrix (OFM) may serve as a suitable biomaterial for this unique application on the basis of existing usage in a range of highly inflamed, irregular, and contaminated wound beds [5]. OFM is a decellularized extracellular matrix (dECM) biomaterial developed for a range of soft tissue repair, reconstruction, and wound healing applications. Once implanted OFM is fully bio-absorbed into the patients regenerating soft tissue and remodeled to leave only functional well-vascularized tissue. OFM-based devices have found utility in the regeneration of contaminated soft tissue defects [5, 6].

To date, there is no reported experience using OFM in PF closure, prompting a single-center retrospective case series to evaluate the use of OFM as a perianal fistula implant (PAFI) to facilitate closure, minimize postoperative complications, and negate the need for more-invasive surgical interventions.

## Methods

The study protocol was reviewed by the LifeBridge Health Institutional Review Board, and ethical oversight of the retrospective study was waived. The study was conducted in accordance with institutional guidelines and the World Medical Association Declaration of Helsinki ethical guidelines. All patient information, including any patient images, were de-identified. All patients signed informed consent for the procedure.

**Fig. 1:**
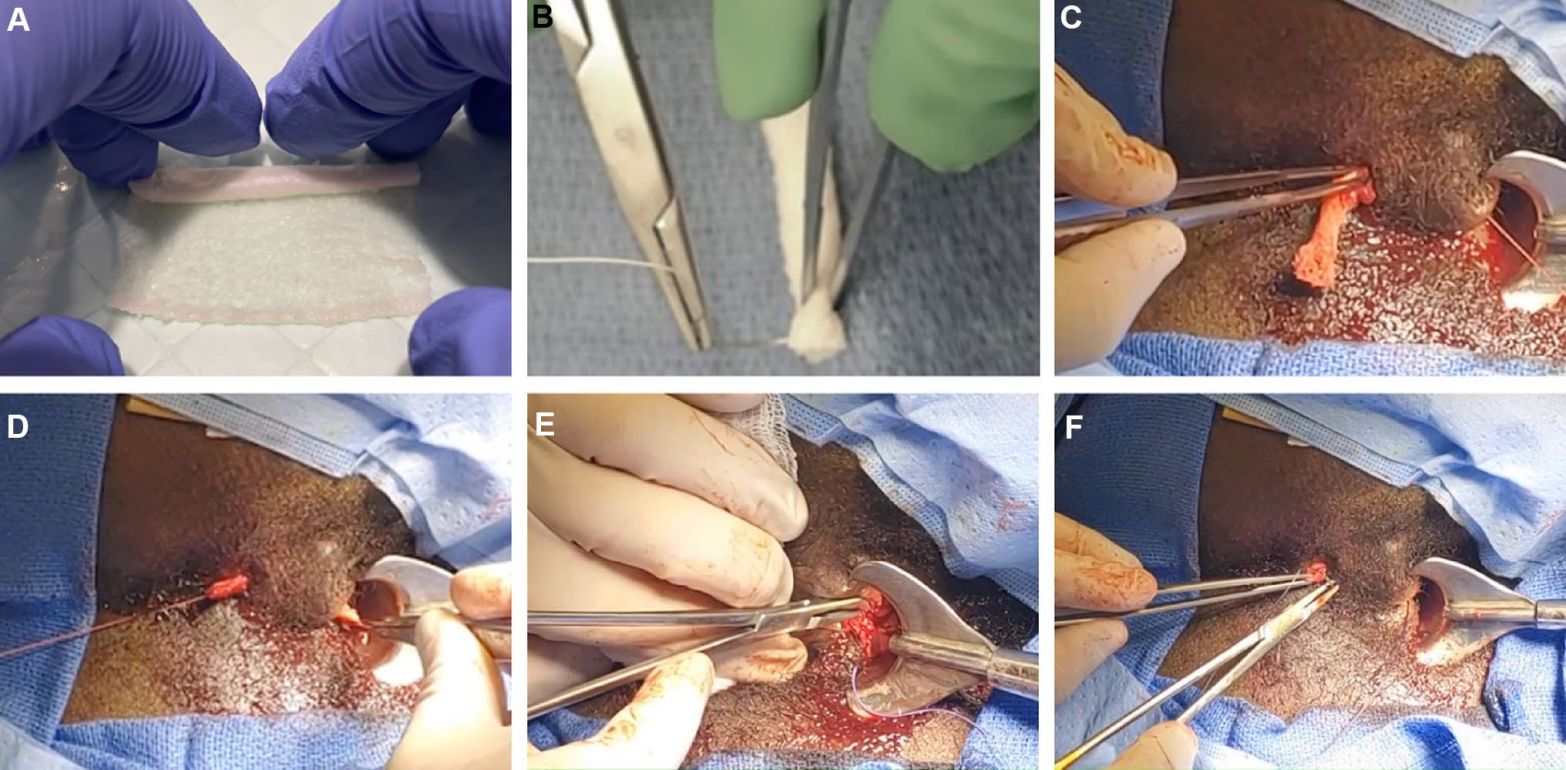
Representative images of the OFM-based PAFI technique. **a** OFM graft (5 × 5 cm) rolled to form a cylindrical implant, approx. 5 × 0.5 cm diameter, then **b** attaching the OFM implant, via suture to the end of a seton. **c** The seton is passed through the PF canal, the OFM implant was drawn through the fistula via the suture aided by forceps. The ends of the OFM implant were then secured internally (**d**) and externally (**e**) with suture

**Fig. 2:**
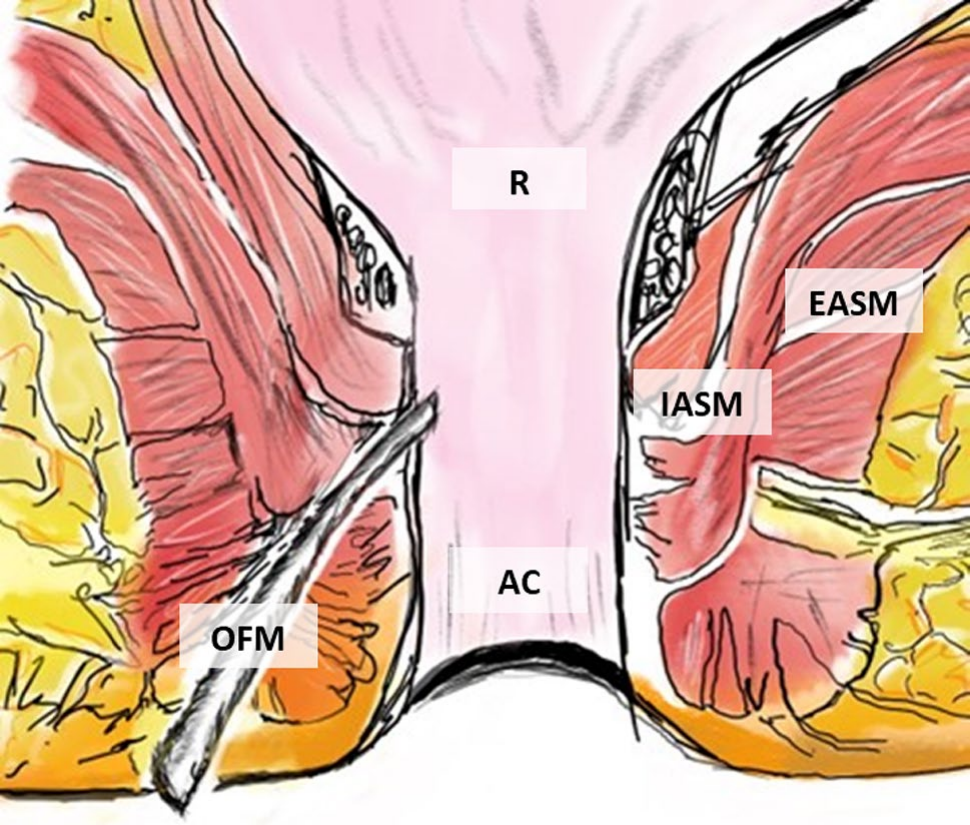
Schematic representation of the OFM-based PAFI technique. *OFM* ovine forestomach matrix implant, *AC* anal canal, *R* rectum, *IASM* internal anal sphincter muscle, *EASM* external anal sphincter muscle

Data were collected from patients that met the inclusion/exclusion criteria (Table 1) and represented consecutive patients that had undergone a minimally invasive PAFI using OFM between November 2020 and February 2023. OFM graft (Myriad Matrix Soft Tissue Bioscaffold™, Aroa Biosurgery Limited, Auckland, New Zealand) was used according to the instructions for use. Fistula tracts were not routinely visualized utilizing magnetic resonance imaging (MRI) as part of standard workup but rather were evaluated via physical examination under anesthesia at the time of diagnosis and/or seton placement. All patients were prepared for fistula closure with a non-cutting seton left in place for at least 12 weeks. If the patient was experiencing persistent purulent discharge from the area of the seton, the PAFI device was not utilized as gross infection is a contraindication of this technique and device. Local anesthesia was administered using 1% lidocaine as a bilateral pudendal and circumferential perianal block. The fistula tract was debrided with a curette to remove the epithelial tissue lining the fistulae. The OFM device (5 × 5 cm, 3-layer) was hydrated with saline, then hand rolled to create a cylindrical implant reflecting the length and diameter of the fistula tract (approx. 4–5 cm × 0.3–0.5 cm) (Fig. 1). The OFM implant was secured to the seton with a Vicryl® suture, and when the seton was removed the OFM implant was pulled into the fistula tract. Once passed, the OFM implant was secured internally and externally with approx. 5 mm of overhang using 2–0 Vicryl® suture (Fig. 2). Additional local anesthesia (0.25% bupivacaine or Exparel®) was administered at case conclusion for pain control. No postoperative dressing was applied, but instead patients were instructed to wear surgical underwear. Follow-up visits were conducted at 2 weeks and 8 weeks, for early follow-up, and then 6 and 12 months for long-term follow-up.

**Table 1:** Inclusion and exclusion criteria

Inclusion criteria	Exclusion criteria
Male or female patients aged 18 years or above Patients with primary or recurrent anal fistula (cryptoglandular disease) treated with OFM as part of their soft tissue reconstruction procedure	Patients still under active management having received their anal fistula treatment < 3 months prior Patients that did not receive OFM as part of their treatment Patients with inflammatory bowel disease Patients with Crohn’s disease Patients with acute perianal infection

Patient demographics (e.g., age, gender, significant baseline comorbidities, Park’s classification), prior surgical interventions, and outcomes (e.g., complete healing, recurrence, complications) were captured in Excel (Microsoft Corporation). Significant patient comorbidities included diabetes mellitus, obesity, psychiatric disorders, atrial fibrillation, diverticulosis, irritable bowel syndrome, and illicit drug use. The primary study outcome was defined as complete healing at postoperative week 8. Secondary endpoints included mean time to complete healing, recurrence of fistula during the follow-up interval, and postoperative complications (e.g., infection, pain, and recurrence). Descriptive statistics (e.g., median, interquartile range (IQR), mean, standard deviation (SD)) were computed using GraphPad Prism (version 9.5.0, Dotmatics Inc).

## Results

A total of ten male and four female participants were included in this case series with a mean age of 56.5 ± 16.0 years (Table 2). Eleven participants presented with additional complicating comorbidities (Table 2). All cases were isolated trans-sphincteric PF, except patient #4 who presented with an additional extra-sphincteric PF. The consecutive case series included five cases presenting as a recurrent PF and nine cases as a primary PF, and *n* = 3/14 (21%) cases had undergone a prior surgical intervention (Table 2). The mean length of the PF was 4.0 ± 0.8 cm, and all participants were experiencing preoperative pain associated with their PF.

**Table 2:** Patient demographics and baseline perianal fistula

Patient #	Age (years)	Male/female	Type of PF	Length (cm)	Primary/recurrent	Prior surgical intervention (yes/no)
1	55	M	Trans-sphincteric	4.0	Recurrent	Yes
2	64	F	Trans-sphincteric	4.0	Recurrent	No
3	74	M	Trans-sphincteric	3.0	Primary	No
4	59	M	Trans-sphincteric Extra-sphincteric	6.0	Primary	Yes
5	60	M	Trans-sphincteric	5.0	Primary	No
6	70	F	Trans-sphincteric	4.0	Recurrent	No
7	66	M	Trans-sphincteric	3.5	Recurrent	No
8	55	M	Trans-sphincteric	4.0	Recurrent	Yes
9	71	M	Trans-sphincteric	3.0	Primary	No
10	50	M	Trans-sphincteric	3.0	Primary	No
11	36	F	Trans-sphincteric	4.0	Primary	No
12	33	F	Trans-sphincteric	4.0	Primary	No
13	23	M	Trans-sphincteric	4.0	Primary	No
14	71	M	Trans-sphincteric	4.0	Primary	No
	56.5 ± 16.0 [56.4 (46.5–70.3)]	71%/29%		4.0 ± 0.8 [4.0, (3.4–4.0)]	64%/36%	21%/79%

Errors represent standard deviation of the mean. Median and IQR are included in [], where applicable

Of the 14 participants, six (43%) were fully healed at week 4 follow-up visit, and nine (64%) were healed at the week 8 visit (Table 3). The week 8 non-healing patients (#5, #8, #10, and #11) experienced drainage from the PF. Two unhealed patients (#5 and #12) at week 8 underwent a second PAFI procedure, with the fistula noted as healed within 4 weeks following the second procedure in both participants. In the case of patient #5, the re-do PAFI was preceded by seton replacement (12 weeks) to de-escalate the inflammation at the fistula site, which had purulent drainage. Patient #12 did not have purulent drainage and the repeat PAFI was performed without seton replacement. There was no significant fibrosis or scarring noted in either of the cases requiring re-implantation. Patient #14 was healed by week 8, but the fistula had recurred at the time of last follow-up visit. Over the study period a total of *n* = 11 (78%) participants had healed, with a median time to fistula closure of 3.6 (IQR 2.9–6.0) weeks (Table 3).

**Table 3:** Study outcomes

Patient #	Outcome at postoperative week 8?	Complications at postoperative week 8?	Postoperative antibiotic use?	Antibiotic duration (days)	Product utilization	Time to heal (weeks)	Last follow-up (weeks)	Recurrence at last follow-up?
1	Healed	None	No	N/A	1	1.6	61.6	No
2	Healed	None	Yes	7	1	3.6	33.3	No
3	Healed	None	No	N/A	1	2.0	42.7	No
4	Healed	None	Yes	7	1	3.3	48.9	No
5	Unhealed	Drainage; repeat PAFI procedure at week 30.9	No	N/A	2	35.7	35.7	No
6	Healed	None	Yes	5	1	2.9	60.3	No
7	Healed	None	Yes	7	1	6.0	45.4	No
8	Unhealed	Drainage	No	N/A	1	–	39.4	N/A
9	Healed	None	No	N/A	1	3.0	35.7	No
10	Unhealed	Drainage	Yes	14	1	–	21.0	N/A
11	Unhealed	Drainage	Yes	7	1	–	15.1	N/A
12	Unhealed	None; repeat procedure at 17.3 weeks	Yes	7	2	21.0	23.9	No
13	Healed	None	Yes	7	1	6.0	8.6	No
14	Healed	None	Yes	7	1	6.0	20.3	Recurrence noted at week 20.3
				7.6 ± 2.5 [7.0, (7.0–7.0)]	1.1 ± 0.4 [1.0, (1.0–1.0)]	9.0 ± 12.8 [3.6, (2.9–6.0)]	37.6 ± 20.1 [35.7, (20.8–50.6)]	

Errors represent standard deviation of the mean. Median and IQR are included in [], where applicable

*N/A* not applicable

## Discussion

The ideal procedure for treatment of PF has been elusive because the invasive closure techniques that have higher efficacy incur higher risk, and minimally invasive techniques with lower risk generally have poor success. PAFI with OFM is a minimally invasive technique that is simple to learn and perform, and in this pilot retrospective cases series resulted in a 64% (*n* = 9/14) healing rate at 8 weeks and 78% (*n* = 11/14) were healed over the study period. There was one recurrent PF (patient #14) in the healed cohort at last follow-up and there were no significant complications in any of the study patients. These findings demonstrate the potential for PAFI with OFM to offer high efficacy with low risk in definitive treatment of PFs.

The use of biologic implants in treating PFs has been described by others previously. dECM and collagen-based implants, termed “fistula plugs”, were first proposed as a minimally invasive alternative to more invasive surgical procedures based on the application of these technologies across a range of soft tissue defects [7]. However, first-generation dECM fistula plugs used in this application had relatively low clinical efficacy compared to invasive surgical procedures. For example, Bondi et al. [1] compared the clinical effectiveness of a porcine small intestine submucosa fistula plug (Surgisis®, Cook Surgical, Bloomington, Indiana, USA) to a mucosal flap procedure, and reported 12-month recurrence rates of 66% and 38%, respectively. These results were consistent with a previous study comparing a dECM fistula plug and an endorectal anal flap (ERAF) procedure, with 12-month recurrence rates of 80% and 12.5%, respectively [8].

It is interesting to speculate on the unique properties of OFM that may contribute to its success in this pilot. Previous studies on OFM have characterized its anti-inflammatory components [9] and in vitro testing has demonstrated its inhibition of tissue proteases [10], key contributors to chronic tissue inflammation [5]. More recently, OFM has been shown to recruit stem cells [9], drawing parallels to the deployment of stem cells in treatment of PF diseases [4]. In addition to the OFM implant being a different source tissue to existing fistula plugs, the OFM implant used in this series was fashioned during the operation, rather than being pre-formed as a plug, and the material can be cut to size. This approach allows for a tailored implant that can be fashioned to fit the dimensions of the patient’s PF, as measured in real time by the operating surgeon. While PF included in the current case series had a length of approx. 4 cm, the approach would also be applicable to shorter PF tracts, though fistula tracts smaller than 2 cm would likely be best treated with a fistulotomy procedure with little to no morbidity. An additional advantage of the method described in this series is the low potential for local tissue disruption compared to other surgical techniques. Techniques that rely upon tissue mobilization and surgical dissection generate fibrosis within the natural tissue planes. In cases of recurrence, this scarring can make subsequent repairs more challenging and may even be prohibitive. In contrast, PAFI carries minimal risk for local scarring and does not compromise or limit subsequent surgical options. As an example from this series, two patients (#5 and #12, Table 3) who failed to heal, underwent a repeat PAFI that resulted in complete healing within 4 weeks of the second procedure. This highlights the relative ease with which the PAFI technique can be deployed, and even in the event of initial failed healing subsequent application may still lead to a successful outcome. This is consistent with wounds of various etiologies which may require multiple applications of extracellular matrix material to achieve healing.

As a retrospective pilot study, there are several limitations to this study. Most importantly, the results are based on a relatively small cohort of patients that were retrospectively reviewed and the follow-up was relatively short. However, on the basis of these initial results, further prospective studies are warranted to validate the results herein. For example, a randomized controlled trial comparing OFM-based PAFI treatment to invasive surgical intervention (e.g., LIFT) or traditional fistula plugs may be considered. Another limitation of the current case series was the absence of MRI characterization of the fistula tracts prior to treatment. Future studies would include MRI evaluations of the PF to aid in diagnosis and evaluation the extent of fistula tracts. The authors are considering prospective study designs to validate and expand the results of this study such as a randomized controlled trials comparing to OFM-based PAFI treatment with standard-of-care or existing PF treatment options such as traditional fistula plugs.

## Conclusion

The promising results of this retrospective pilot case series suggest that an OFM implant may be a clinically successful and minimally invasive treatment option for the treatment of PF. 

## Supplementary Information

Below is the link to the electronic supplementary material.

Supplementary file1 (MP4 463685 KB)

## Data Availability

All data generated or analysed during this study are included in this published article [and its supplementary information files].
